# Remote Ischemic Preconditioning Reduces Marathon-Induced Oxidative Stress and Decreases Liver and Heart Injury Markers in the Serum

**DOI:** 10.3389/fphys.2021.731889

**Published:** 2021-09-06

**Authors:** Jan Mieszkowski, Błaz∙ej Stankiewicz, Andrzej Kochanowicz, Bartłomiej Niespodziński, Andz∙elika Borkowska, Katarzyna Sikorska, Ludmiła Daniłowicz-Szymanowicz, Paulina Brzezińska, Jędrzej Antosiewicz

**Affiliations:** ^1^Department of Gymnastics and Dance, Gdansk University of Physical Education and Sport, Gdańsk, Poland; ^2^Faculty of Physical Education and Sport, Charles University, Prague, Czechia; ^3^Department of Human Biology, Institute of Physical Education, Kazimierz Wielki University, Bydgoszcz, Poland; ^4^Department of Bioenergetics and Physiology of Exercise, Faculty of Health Sciences, Medical University of Gdańsk, Gdańsk, Poland; ^5^Department of Tropical and Parasitic Diseases, Faculty of Health Sciences, Medical University of Gdańsk, Gdańsk, Poland; ^6^Department of Cardiology and Electrotherapy, Medical University of Gdańsk, Gdańsk, Poland

**Keywords:** skeletal muscle damage, creatinine kinase MB, malondialdehyde, remote ischemic preconditioning, inflammation

## Abstract

Clinical studies continue to provide evidence of organ protection by remote ischemic preconditioning (RIPC). However, there is lack of insight into impact of RIPC on exercise-induce changes in human organs’ function. We here aimed to elucidate the effects of 10-day RIPC training on marathon-induced changes in the levels of serum markers of oxidative stress, and liver and heart damage. The study involved 18 male amateur runners taking part in a marathon. RIPC training was performed in the course of four cycles, by inflating and deflating a blood pressure cuff at 5-min intervals (RIPC group, *n*=10); the control group underwent sham training (*n*=8). The effects of RIPC on levels of oxidative stress, and liver and heart damage markers were investigated at rest after 10 consecutive days of training and after the marathon run. The 10-day RIPC training decreased the serum resting levels of C-reactive protein (CRP), alanine transaminase (ALT), γ-glutamyl transpeptidase (GGT), and malondialdehyde (MDA). After the marathon run, creatinine kinase MB (CK-MB), lactate dehydrogenase (LDH), cardiac troponin level (cTn), aspartate aminotransferase (AST), alkaline phosphatase (ALP), ALT, total bilirubin (BIL-T), and MDA levels were increased and arterial ketone body ratio (AKBR) levels were decreased in all participants. The changes were significantly diminished in the RIPC group compared with the control group. The GGT activity remained constant in the RIPC group but significantly increased in the control group after the marathon run. In conclusion, the study provides evidence for a protective effect of RIPC against liver and heart damage induced by strenuous exercise, such as the marathon.

## Introduction

Prolonged strenuous running, e.g., a marathon run, induces a rise in the concentrations and/or activity of biomarkers that reflect physiological stress of the skeletal muscle, liver, heart, and some other tissues (Banfi et al., 2012). Oxidative stress is one of the processes associated with tissue damage. It can be induced by exercise, especially by prolonged forms of exercise, such as the marathon and its more demanding variations (Gomez-Cabrera et al., 2006; Kawamura and Muraoka, 2018). Consequently, high-intensity or long-duration exercise can potentially lead to major changes in the markers of tissue damage that are commonly associated with pathological states (Smith et al., 2004). For instance, the concentration of serum cardiac troponin T (cTnT), one of the markers of heart muscle damage, increase after endurance events in up to 68%, and reach levels typically diagnostic of acute myocardial infarction (Fortescue et al., 2007; Eijsvogels et al., 2016). Similarly, after a long-distance run, liver injury biomarkers, such as aspartate aminotransferase (AST), γ-glutamyl transpeptidase (GGT), and lactate dehydrogenase (LDH) activities, and conjugated bilirubin, are elevated (Lippi et al., 2011; Shin et al., 2016). Changes in the concentration and/or activity of markers of heart and liver damage after a marathon run are transient and most return to the baseline after several days. While there is insufficient evidence to indicate any adverse effects of exercise on the heart (Kaleta-Duss et al., 2020) and liver (Lippi et al., 2011) of amateur marathon runners, further studies investigating the impact of strenuous exercise on the markers of heart and liver damage can give insight into the physiology of adaptation of these organs to a prolonged strenuous exercise.

Multiple studies aim to identify the optimal approach of reducing the impact of strenuous exercise on markers of tissue damage (for review see Kawamura and Muraoka, 2018). Currently, increasing attention is being focused on remote ischemic preconditioning (RIPC), which has been shown to be protective against ischemia-reperfusion injury (Tapuria et al., 2008) and other stressors, and can potentially increase sports performance (Caru et al., 2019). RIPC is a procedure, whereby brief cycles of limb ischemia and reperfusion are induced by inflating and deflating a blood pressure cuff. In this manner, skeletal muscle undergoes preconditioning and distal tissues are protected. RIPC reduces the increase of cardiac troponin levels (cTn) by 42% after coronary artery bypass (Venugopal et al., 2009), and by approximately 30% after valve replacement surgery (Cao et al., 2017). Similarly, it was shown that RIPC, when applied before liver resection, decreases the AST activity and bilirubin levels in comparison to control, not pre-conditioned, patients (Rakic et al., 2018; Liu et al., 2019; Wu et al., 2020). A similar trend was observed for RIPC and liver transplants (Robertson et al., 2017). Further, RIPC effectively reduces oxidative stress in some clinical scenarios, e.g., cardiopulmonary bypass and others (Chen et al., 2015; Arvola et al., 2016; Pinheiro et al., 2016) and improve peripheral endothelial function (Maxwell et al., 2019).

The reported outcomes indicate that RIPC might impact exercise-induce changes in the markers of heart and liver damage. However, to date, the data on this topic are limited and conflicting (El Messaoudi et al., 2013; Cocking et al., 2017). In the current study, we aimed to determine the effects of RIPC on the markers of heart and liver damage, and oxidative stress induced by a marathon run in amateur runners.

## Materials and Methods

### Ethics Statement

The study was approved by the Bioethics Committee for Clinical Research at the Regional Medical Chamber in Gdańsk (decision number KB-24/16; Gdańsk, Poland) and was conducted in accordance with the declaration of Helsinki. Written informed consent was obtained from all participants before inclusion in the study. The participants were informed about the possibility of withdrawing their consent at any time and for any reason, and were informed about the study procedures, but not about the rationale and study aim, so as to keep them naive as to the potential effects of RIPC.

### Experimental Overview

In the study, the effects of 10-day RIPC training on marathon-induced changes in the markers of oxidative stress, and liver and heart damage were evaluated. All participants were randomly assigned to two study groups undergoing either RIPC or sham-controlled intervention (RIPC vs. SHAM) for 10 consecutive days. During the first visit (pre-intervention) to laboratory, set early in the morning, basic anthropometric characteristics were measured (the subject’s age, body composition, and height) and venous blood samples were drawn. On the following and subsequent days, either RIPC or SHAM procedure took place. Early in the morning 1day after the last RIPC or SHAM training, blood samples were collected; the runners performed a marathon race on the same day. Blood samples were also collected immediately after, 24h after, and 7days after finishing the race.

### Participants

Twenty-four male amateur runners were enrolled in the study: 12 in the RIPC group and 12 in the SHAM group. Each runner had an experience of a minimum five full marathon runs, with the completion time between 2h 50min and 3h 20min. For the study, each participant ran the 46th Dębno Marathon (Dębno, West Pomerania Province, Poland). The starting temperature on the day was 12.1°C, and the starting (and finishing) line was in the town of Dębno (40m above sea level). The course is flat and allows the runners to achieve high running speed.

Two runners from the RIPC group and four from the SHAM group did not finish the race. Consequently, data for only 18 amateur runners were analyzed (RIPC, *n*=10; SHAM, *n*=8). The basic anthropometric characteristics of the groups and their performance are shown in Table 1. Before the experiment, a physician examined all the participants, and confirmed that they were healthy, with no history of known diseases that may have affected the physical performance (examination – blood pressure, heart rate, ECG, and respiratory parameters). From the time of enrolment to the end of the study period, none of the runners reported intake of any medication or drugs, and refrained from alcohol, caffeine, guarana, theine, tea, and chocolate, as these substances may potentially influence exercise performance. Upon enrolment, the participants have adopted similar eating patterns, based on a randomized diet for their age group and physical intensity. Each participants completed survey aimed to define the training loads used during training period (divided into a periods of general preparation – 3months and pre-start preparation – 2months before the run; Supplementary Material).

**Table 1:** Physical characteristics and the marathon run performance of the participants (mean±SD).

Variable	Overall (*n*=19)	RIPC (*n*=10)	SHAM (*n*=9)	RIPC vs. SHAM (*p*)
Age (year)	36.05±3.25	36.70±3.57	35.33±2.66	0.39
Body mass (kg)	76.36±7.16	72.60±7.14	76.44±2.66	0.16
Height (cm)	182.52±3.11	182.60±3.95	182.44±1.77	0.91
Body mass index (kg/m^2^)	22.91±1.97	21.77±1.60	22.96±1.05	0.08
Average running speed (km/h)	11.85±0.66	12.14±0.57	11.57±0.64	0.08
Average running time (min)	213.57±12.76	208.9±10.45	218±13.32	0.09

RIPC, remote ischemic preconditioning training group; and SHAM, sham-controlled group.

### RIPC Procedures

Each participant underwent 10 consecutive days (Thijssen et al., 2016) of either RIPC or SHAM conditioning before the marathon run. In both cases, the procedure was performed in the supine position, with bilateral arterial occlusion of both legs (Griffin et al., 2018; Mieszkowski et al., 2020). The occlusion cuff was positioned proximally around the thigh and inflated to 220mmHg (to block the arterial inflow) or 20mmHg (placebo effect) in the RIPC and SHAM groups, respectively (Paull and Van Guilder, 2019; Mieszkowski et al., 2020). Both procedures consisted of four sets of 5-min inflation, followed by 5-min deflation (Cocking et al., 2018).

The RIPC or SHAM procedure was performed at the same time (early morning) each day and under the control of color flow Doppler ultrasound (Edan DUS 60; Edan Instruments GmbH SonoTrax Basic, Langen, Germany) to ensure the full closure of the arterial inflow. All ultrasound procedures were performed according to the standards of the Polish Ultrasound Society, by a physician who had completed a training in ultrasound imaging. The participants had no knowledge of the group allocation and differences in the procedures.

### Sample Collection and Inflammation Marker Determinations

The blood was collected five times: before and 24h after the RIPC/SHAM training period (latter served also as before the marathon run measurement), immediately after, and 24h and 7days after the marathon race. Venous blood samples were collected into Sarstedt S-Monovette tubes (S-Monovette® Sarstedt AG&Co, Nümbrecht, Germany) containing a coagulant for blood analysis; into tubes without anticoagulant for serum separation (with coagulation accelerator); or in tubes containing EDTA for plasma isolation. Samples were centrifuged using standard laboratory methods, aliquoted, and frozen at −80°C until further analysis. The selected markers were analyzed according to the medical diagnostic procedures referenced by European Federation of Clinical Chemistry and Laboratory Medicine. EFLM by Synevo Labolatory at an accredited laboratory (Bydgoszcz, Poland; PN-EN ISO 15189) using a hematological analyzer, and immunoenzymatic and conductometric methods, as appropriate (Sysmex XS-1000i apparatus, Roche/Hitachi Cobas c. system using a Cobas c 501 analyzer, Thermo Scientific Multiscan GO Microplate Spectrophotometer produced by Fisher Scientific Finland).

The following were analyzed: (1) cardiovascular and cardiac muscle markers: creatinine kinase (CK), creatinine kinase MB (CK-MB), C-reactive protein (CRP), hemoglobin (HGB), LDH activity, myoglobin (MB), red blood cells (RBC), troponin T (TnT), and urea; (2) liver markers: albumin (ALB), alkaline phosphatase (ALP) activity, alanine transaminase (ALT) activity, AST activity, direct bilirubin (BIL-D), total bilirubin (BIL-T), GGT activity, globulin (GLB), and total protein (TP); and (3) oxidative stress markers: malondialdehyde (MDA), arterial ketone body ratio (AKBR), and conjugated dienes (CD).

### Statistical Analysis

Descriptive statistics (the mean±SD) were used for all measured variables. Two-way ANOVA with repeated measures (*group*: RIPC, SHAM×*training*: before, after) was used to investigate the difference between the effects of 10-day RIPC and SHAM training on the selected cardiac muscle, liver, and oxidative stress markers. Another two-way ANOVA with repeated measures (*group*: RIPC, SHAM; *marathon*: before, immediately after, and 24h and 7days after the marathon) was performed to investigate the impact of marathon running on the selected marker levels in relation to the preceding 10days of RIPC training. In case of a significant interaction, Tukey’s *post hoc* test was performed to assess differences in particular subgroups. In addition, the effect size was estimated by eta-squared statistics (*ƞ*^2^). Values equal to or greater than 0.01, 0.06, and 0.14 indicated a small, moderate, and large effect, respectively. All calculations and graphics were done in Statistica 12 software (StatSoft, Tulsa, OK, United States). Differences were considered statistically significant at *p*≤0.05. The required sample size was estimated by using GPower ver. 3.19.4 software (Faul et al., 2007). The power analysis for interaction between analyzed factors in two-way ANOVA of repeated measures show the minimal total sample sized for the large effect size with power of 0.95 was equal to 16 subjects.

## Results

### Effects of 10-Day RIPC Training on the Markers of Heart and Liver Damage and Oxidative Stress

The effects of 10-day RIPC training on the biomarkers of heart and liver damage, and markers of oxidative stress at rest are shown in Table 2.

**Table 2:** Changes in cardiovascular and muscle damage, hepatic and oxidative stress associated biomarkers induced by 10-day remote ischemic preconditioning training.

Biomarkers	Variable	RIPC group		SHAM group	
		Pre	Post	Pre	Post
		Mean±SD	Mean±SD	Mean±SD	Mean±SD
Cardiovascular and muscle damage	BUN (mmol/L)	5.79±0.79	5.37±1.04^*^	6.05±0.69	5.63±0.98^*^
	CK (U/L)	107.70±27.68	80.30±35.24^*^	115.42±15.39	93.87±17.92^*^
	CK-MB (ng/ml)	3.71±0.54	2.95±1.04^*^	3.77±0.31	3.47±0.43^*^
	CRP (mg/L)	2.92±0.72	1.97±0.92^*^	2.71±0.65	3.03±1.11
	HGB (g/dl)	15.29±0.83	15.42±0.80	14.74±0.57	14.85±0.60
	LDH (U/L)	263.08±39.20	249.40±43.13^*^	266.96±29.91	237.26±33.92^*^
	MB (ng/ml)	95.20±20.32	71.80±12.60^*^	94.65±7.35	57.79±16.08^*^
	RBC (×10^6^/μl)	4.81±0.28	4.95±0.28^*^	4.53±0.27	4.75±0.32^*^
	TnT (ng/ml)	0.51±0.50	0.40±0.69	0.70±0.45	0.49±0.17
Hepatic	ALB (g/L)	41.16±0.78	41.55±0.88^*^	43.21±1.17	43.30±0.95^*^
	ALP (U/L)	132.44±2.42	129.48±1.96^*^	135.22±6.67	131.96±4.98^*^
	ALT (U/L)	29.00±3.05	26.20±2.89^*^	29.79±2.70	29.03±4.03
	AST (U/L)	28.61±6.04	27.30±6.64^*^	31.39±2.29	29.34±4.56^*^
	BIL-D (μmol/L)	2.67±0.21	2.52±0.20^*^	2.93±0.43	2.72±0.21^*^
	BIL-T (μmol/L)	12.43±0.43	11.65±0.57^*^	12.42±0.59	11.42±0.59^*^
	GGT (U/L)	44.00±8.08	38.30±7.68^*^ ^#^	53.12±6.10	51.14±6.21
	GLB (g/L)	29.73±1.44	29.45±1.35	28.87±0.69	28.60±0.86
	TP (g/L)	71.22±1.02	71.59±0.77	72.11±0.87	71.99±0.84
Oxidative stress	AKBR	0.71±0.06^#^	0.90±0.06^*^ ^#^	0.67±0.05	0.80±0.05^*^
	CD (abs/ml)	15.58±1.88^#^	9.82±2.36^*^ ^#^	17.23±1.70	12.98±0.88^*^
	MDA (μmol/L)	0.42±0.18	0.07±0.05^*^ ^#^	0.45±0.10	0.25±0.06^*^

RIPC, 10days of remote ischemic preconditioning; SHAM, 10days of sham controlled intervention; BUN, blood urea nitrogen; CK, creatine kinase; CK-MB, creatinine kinase MB; CRP, c-reactive protein; HGB, hemoglobin, LDH, lactate dehydrogenase; MB, myoglobin; RBC, red blood cells; TnT, troponin T; ALB, albumin; ALP, alkaline phosphatase; ALT, alanine aminotransferase; AST, aspartate aminotransferase; BIL-D, direct bilirubin; BIL-T, total bilirubin; GGT, γ-glutamyl transpeptidase; GLB, globulin; TP, total protein; MDA, malondialdehyde; AKBR, arterial ketone index; and CD, conjugated dienes.

* Significant difference vs. pre at p < 0.01;

# Significant difference vs. SHAM at p < 0.01.

Two-way ANOVA revealed that the levels of CK, CK-MB, LDH activity, MB, urea, ALP activity, BIL-T, BIL-D, AST activity, and CD decreased after 10days of training irrespective of RIPC. By contrast, AKBR, ALB, and RBC levels increased after 10days in both groups. The effect of RIPC training was only noted for CRP levels, and ALT and GGT activity, wherein the resting values of these markers decreased and those in the SHAM group remained unchanged. However, only the changes in GGT activity levels were significantly different between the groups. Further, while MDA levels were reduced in both groups, the RIPC training led to a significantly greater reduction in the levels of this marker than SHAM training. Of note, while the levels of most of the investigated markers did not differ between the groups before the intervention, the pre-training AKBR and CD levels were significantly higher and lower, respectively, in the RIPC group than those in the SHAM group. These differences were maintained after 10days of training.

### Effect of 10-Day RIPC Training on Changes in Marathon-Induced Markers of Cardiovascular System and Heart Damage

**Figure 1:**
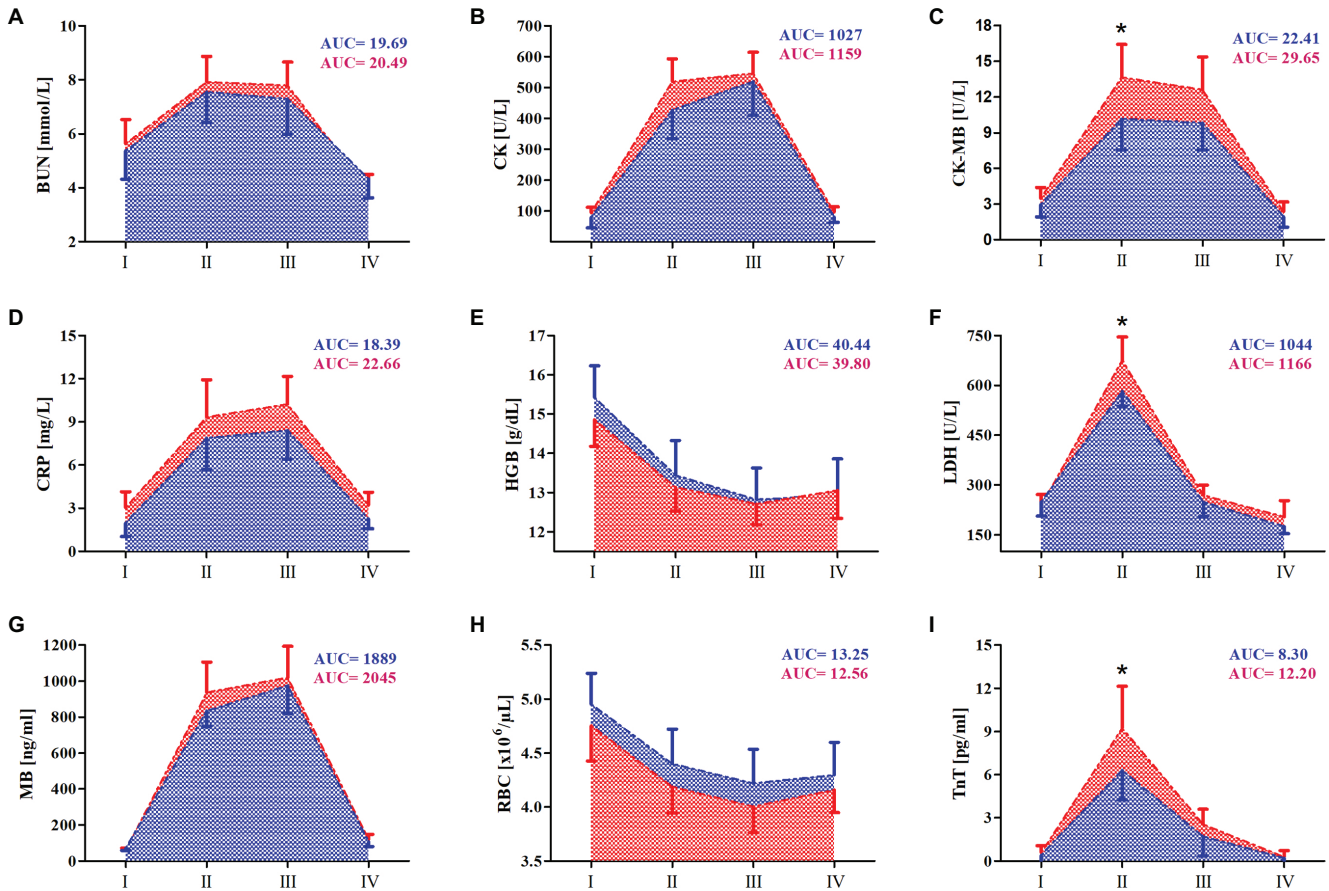
Change in serum of selected hearth damage and cardiovascular markers after marathon run. **(A)** Blood urea nitrogen (BUN), **(B)** creatinine kinase (CK), **(C)** creatinine kinase MB (CK-MB), **(D)** C-reactive protein (CRP), **(E)** hemoglobin (HGB), **(F)** lactate dehydrogenase (LDH), **(G)** myoglobin (MB), **(H)** red blood cells (RBCs), and **(I)** troponin T (TnT). Blue color – a group after 10days remote ischemic preconditioning training (RIPC), red color – a group after sham-controlled intervention (SHAM), AUC, area under curve, I – before the marathon run (baseline), II – immediately after marathon run, III – 24h, and IV – 7days after marathon. ^*^Significant difference vs. RIPC at *p*<0.01.

Changes in the levels of markers of heart damage following the marathon run in relation to RIPC training are presented in Figure 1.

The results of two-way ANOVA are shown in Table 3. The levels of the following markers increased in both groups immediately after the marathon run: CK, CK-MB, CRP, LDH activity, MB, TnT, and urea. The increase persisted up to 24h after the run, except for the TnT and LDH activity levels, which returned to the approximate resting levels a day after the run. Further, the RBC and HGB levels decreased immediately after the marathon run and the reduction persisted for up to 7days after the run. The effect of the RIPC training on TnT, CK-MB, and LDH activity levels was apparent immediately after the marathon run, in that RIPC attenuated the increase of the levels of heat damage markers (Figure 1).

**Table 3:** Two-way (two groups×four repeated measurements) ANOVA tests for the cardiovascular and muscle damage markers induced by marathon run in RIPC and SHAM groups.

Variable	Effect	*F*	df	*p*	Effect size (*η*^2^)	*Post hoc* outcome
BUN	Group	0.23	1, 16	0.63	0.01	
	Marathon	115.28	3, 48	0.01^*^	0.87	II, III>I, IV; IV<I, II, III
	Group×Marathon	1.73	3, 48	0.17	0.09	
CK	Group	1.53	1, 16	0.23	0.08	
	Marathon	203.05	3, 48	0.01^**^	0.92	III>I, II, IV; II>I, IV
	Group×Marathon	1.22	3, 48	0.31	0.07	
CK-MB	Group	6.07	1, 16	0.02^*^	0.27	RIPC<SHAM
	Marathon	281.30	3, 48	0.01^**^	0.94	II, III>I, IV
	Group×Marathon	6.56	3, 48	0.01^**^	0.29	II, III-IPC>I, IV-RIPC
						II, III-SHAM>I, IV-SHAM
						II-RIPC<II-SHAM
CRP	Group	4.21	1, 16	0.05^*^	0.21	
	Marathon	185.98	3, 48	0.01^**^	0.92	I<II, III; IV>II, III
	Group×Marathon	0.51	3, 48	0.67	0.03	
HGB	Group	0.48	1, 16	0.49	0.02	
	Marathon	94.53	3, 48	0.01^**^	0.82	I>II, III, IV; II>III
	Group×Marathon	1.68	3, 48	0.18	0.11	
LDH	Group	5.21	1, 16	0.03^*^	0.24	RIPC<SHAM
	Marathon	501.59	3, 48	0.01^**^	0.96	II>I, III, IV; IV<I. II, III
	Group×Marathon	6.20	3, 48	0.01^**^	0.27	II-RIPC>I, III, IV-RIPC
						IV-RIPC<I,III-RIPC
						II-SHAM>I, III, IV-SHAM
						II-RIPC<II-SHAM
MB	Group	1.06	1, 16	0.31	0.06	
	Marathon	576,29	3, 48	0.01^**^	0.97	III>I, II, IV; II>I, IV
	Group×Marathon	1.46	3, 48	0.23	0.08	
RBC	Group	1.60	1, 16	0.22	0.09	
	Marathon	60.70	3, 48	0.01^**^	0.79	I>II, III, IV; II>III
	Group×Marathon	0.17	3, 48	0.91	0.01	
TnT	Group	5.04	1, 16	0.04^*^	0.23	RIPC<SHAM
	Marathon	117.93	3, 48	0.01^**^	0.87	II>I, III, IV; III>I, IV
	Group×Marathon	4.20	3, 48	0.01^*^	0.20	II-RIPC>I, III, IV-RIPC
						II-SHAM>I, III, IV-SHAM
						II-RIPC<II-SHAM

RIPC, group after 10days of remote ischemic preconditioning; SHAM, group after 10days of sham controlled intervention; BUN, blood urea nitrogen; CK, creatinine kinase; CK-MB, creatinine kinase MB; CRP, C-reactive protein; HGB, hemoglobin; LDH, lactate dehydrogenase; RBC, red blood cells; TnT, troponin T; and I, before, II, immediately after, III, 24h after, and IV, 7days after the marathon run.

* Significant difference at *p*<0.05;

** Significant difference at *p*<0.01.

### Effect of 10-Day RIPC Training on Changes in Marathon-Induced Markers of Liver Damage

**Figure 2:**
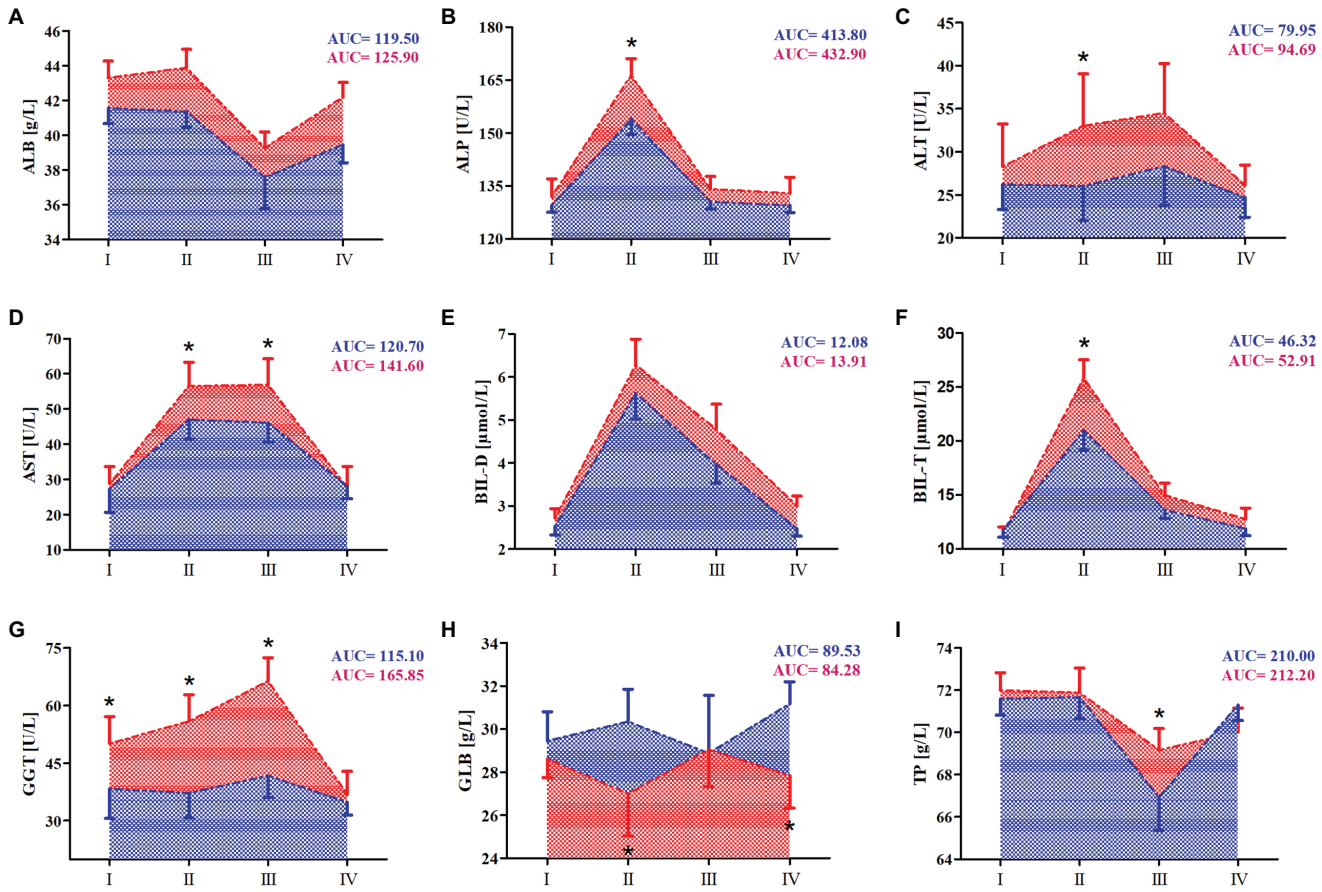
Change in serum of selected hepatic markers after marathon run. **(A)** Albumin (ALB), **(B)** alkaline phosphatase (ALP), **(C)** alanine transaminase (ALT), **(D)** aspartate aminotransferase (AST), **(E)** direct bilirubin (BIL-D), **(F)** total bilirubin (BIL-T), **(G)** γ-glutamyl transpeptidase (GGT), **(H)** globulin (GLB), and **(I)** total protein (TP). Blue color – a group after 10days RIPC training, Red color – a group after sham-controlled intervention (SHAM), AUC, area under curve, I – before the marathon run (baseline), II – immediately after marathon run, III – 24h, and IV – 7days after marathon. ^*^Significant difference vs. RIPC at *p*<0.01.

The effects of RIPC training on the markers of liver damage before and after the marathon run are shown in Figure 2. The results of two-way ANOVA are shown in Table 4. ALP, BIL-D, BIL-T, and AST activity levels increased in both groups immediately after the run. In the RIPC group, the increase of ALP and AST activity, and BIL-T levels was smaller than that in the SHAM group. For ALT and GGT activity, after the marathon run, these markers only increased in the SHAM group and they remained at resting level in the RIPC group. Of note, GGT activity in the SHAM group was elevated before the marathon run and decreased to below the resting level 7days after the run. By contrast, 24h after the run, a decrease in ALB and TP levels was observed in both groups. The decrease was more pronounced in the RIPC group than in the SHAM group. Further, TP levels returned to resting values within next 7days in the RIPC group but not in the SHAM group. Finally, compared with the RIPC group, GLB levels decreased immediately after and 7days after the run in the SHAM group.

**Table 4:** Two-way (two groups×four repeated measurements) ANOVA tests for the hepatic markers induced by marathon run in RIPC and SHAM groups.

Variable	Effect	*F*	df	*p*	Effect size (*η*^2^)	*Post hoc* outcome
Albumin	Group	24.71	1, 16	0.01^**^	0.60	RIPC<SHAM
	Marathon	135.03	3, 48	0.01^**^	0.89	III<I, II, IV; IV<I, II
	Group×Marathon	2.57	3, 48	0.06	0.13	
ALP	Group	30.01	1, 16	0.01^**^	0.65	RIPC<SHAM
	Marathon	319.38	3, 48	0.01^**^	0.95	II>I, III, IV
	Group×Marathon	8.40	3, 48	0.01^**^	0.34	II-RIPC>I, III, IV-RIPC
						II-SHAM>I, III, IV-SHAM
						II-RIPC<II-SHAM
ALT	Group	5.72	1, 16	0.03^*^	0.26	RIPC<SHAM
	Marathon	20.05	3, 48	0.01^**^	0.55	III>I, IV; II>IV
	Group×Marathon	5.90	3, 48	0.01^**^	0.26	III-RIPC>IV-RIPC
						II, III-SHAM>I, IV-SHAM
						II-RIPC<II-SHAM
AST	Group	6.51	1, 16	0.02^*^	0.29	RIPC<SHAM
	Marathon	168.68	3, 48	0.01^**^	0.91	II, III>I, IV
	Group×Marathon	7.18	3, 48	0.01^**^	0.31	II, III-RIPC>I, IV-RIPC
						II, III-SHAM>I, IV-SHAM
						II, III-RIPC<II, III-SHAM
BIL-D	Group	21.45	1, 16	0.01^**^	0.57	RIPC<SHAM
	Marathon	298.73	3, 48	0.01^**^	0.94	II>I, III, IV; III>I, IV
	Group×Marathon	2.12	3, 48	0.10	0.11	
BIL-T	Group	24.12	1, 16	0.01^**^	0.60	RIPC<SHAM
	Marathon	947.61	3, 48	0.01^**^	0.98	II>I,III, IV; I<III, VI
	Group×Marathon	38.17	3, 48	0.01^**^	0.70	II-RIPC>I, III, IV-RIPC
						I-RIPC<III-RIPC
						II-SHAM>I, III, IV-SHAM
						I-SHAM<III, IV-SHAM
						II-RIPC<II-SHAM
GGT	Group	30.07	1, 16	0.01^**^	0.65	RIPC<SHAM
	Marathon	88.02	3, 48	0.01^**^	0.84	III>I, II, IV; IV<I, II
	Group×Marathon	36.87	3, 48	0.01^**^	0.69	III-SHAM>I, II, IV SHAM
						IV-SHAM<I, II-SHAM
						I, II-RIPC<I, II-SHAM
						I, II, III-RIPC<I, II, III-SHAM
Globulin	Group	10.46	1, 16	0.01^**^	0.39	RIPC>SHAM
	Marathon	1.45	3, 48	0.23	0.08	
	Group×Marathon	6.42	3, 48	0.01^**^	0.28	III-RIPC>IV-RIPC
						II-RIPC>II-SHAM
						IV-RIPC>IV-SHAM
Total protein	Group	1.20	1, 16	0.29	0.07	
	Marathon	74.90	3, 48	0.01^**^	0.82	I, II>III, IV; III<IV
	Group×Marathon	12.50	3, 48	0.01^**^	0.43	I, II-RIPC>III-RIPC
						IV-RIPC<III-RIPC
						I, II-SHAM>III, IV-SHAM
						III-RIPC<III-SHAM

RIPC, group after 10days of remote ischemic preconditioning; SHAM, group after 10days of sham controlled intervention; ALP, alkaline phosphatase; ALT, alanine transaminase; AST, aspartate aminotransferase; BIL-T, total bilirubin; BIL-D, direct bilirubin, GGT, γ-glutamyl transpeptidase; and I, before; II, immediately after; III, 24h after; and IV, 7days after the marathon run.

* Significant difference at *p*<0.05;

** Significant difference at *p*<0.01.

### Effect of 10-Day RIPC Training on Changes in Marathon-Induced Markers of Oxidative Stress

**Figure 3:**
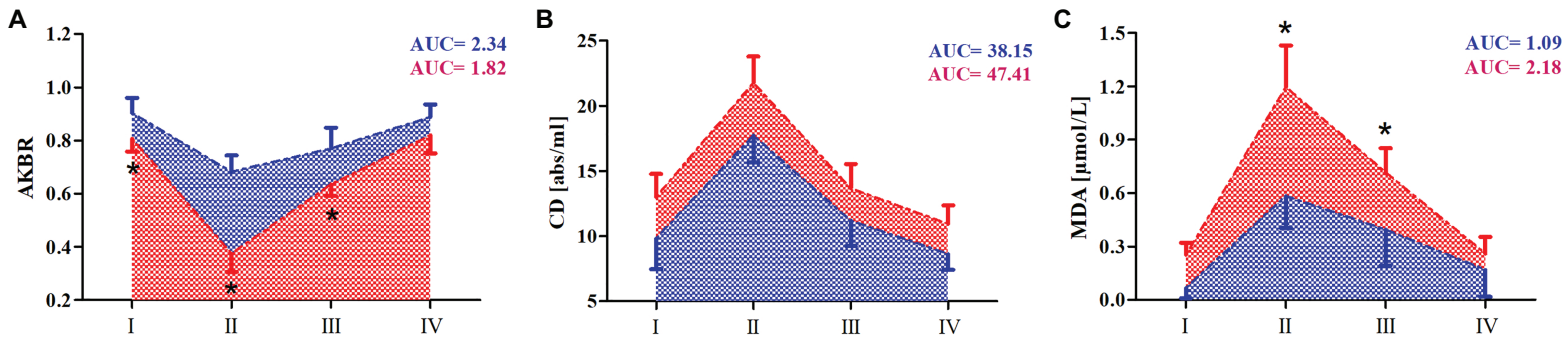
Marathon-induced changes in markers of oxidative stress in RIPC and SHAM groups. **(A)** Arterial ketone body ratio (AKBR), **(B)** conjugated dienes (CD), and **(C)** malondialdehyde (MDA). Blue color – a group after 10 days RIPC, red color – a group after sham-controlled intervention (SHAM), AUC, area under curve, I – before, II – immediately after, III – 24h after, and IV – 7days after the marathon run. ^*^Significant difference vs. RIPC at *p*<0.01.

To evaluate the effect of RIPC training on free radical generation in response to marathon run, three markers of oxidative stress were determined in the athlete serum: AKBR, MDA, and CD (Figure 3). The results of two-way ANOVA are shown in Table 5.

**Table 5:** Two-way (two groups×four repeated measurements) ANOVA tests for the oxidative stress markers induced by marathon run in RIPC and SHAM groups.

Variable	Effect	*F*	df	*p*	Effect size (*η*^2^)	*Post hoc* outcome
AKBR	Group	69.71	1, 16	0.01^*^	0.81	RIPC>SHAM
	Marathon	144.18	1, 16	0.01^*^	0.90	I, IV>II, III
	Group×Marathon	17.14	1, 16	0.01^*^	0.51	I, IV-RIPC>II, III-RIPC
						II-RIPC<III-RIPC
						II-SHAM<I, III, IV-SHAM
						III-SHAM<I, IV-SHAM
						I-RIPC>I-SHAM
						II-RIPC>II-SHAM III-RIPC>III-SHAM
CD	Group	23.64	1, 16	0.01^*^	0.59	RIPC<SHAM
	Marathon	220.03	1, 16	0.01^*^	0.93	II>I, III, IV; IV<I, III
	Group×Marathon	1.77	1, 16	0.16	0.09	
MDA	Group	28.86	1, 16	0.01^*^	0.64	RIPC<SHAM
	Marathon	168.12	1, 16	0.01^*^	0.91	II>I, III, IV; III>I, IV
	Group×Marathon	18.77	1, 16	0.01^*^	0.53	II, III-RIPC>I, IV-IPC
						II-RIPC>III-RIPC
						II, III-SHAM>I, IV-SHAM
						II-SHAM>III-SHAM
						II-RIPC<II-SHAM III-RIPC<III-SHAM

RIPC, group after 10days of remote ischemic preconditioning; SHAM, group after 10days of sham controlled intervention; AKBR, arterial ketone body ratio; CD, conjugated dienes; MDA, malondialdehyde; and I, before, II, immediately after, III, 24h after, and IV, 7days after the marathon run. Significant difference at p < *0.01.

Immediately after and 24h after the marathon run, the CD and MDA levels increased in both groups. The increase of MDA levels was smaller in the RIPC group than that in the SHAM group. Further, the CD levels were significantly lower in the RIPC group than in the SHAM group at all time points measured, showing no interaction with the marathon run. In turn, the AKBR levels decreased immediately after and 24h after the marathon run in both groups. The reduction of AKBR levels was more pronounced in the SHAM group than in the RIPC group; however, the resting levels of this marker were lower in the SHAM group than those in the RIPC group.

## Discussion

The main goal of the current study was to evaluate the effects of 10-day RIPC training on serum biomarkers of liver and heart damage induced by a marathon run. The observed significant increase in the levels of TnT and CK-MB, the markers of heart damage, was attenuated in runners who underwent RIPC training prior to the run. A similar effect of RIPC was observed on the levels of ALT, AST, and GGT activity, and BIL-T, the markers of liver damage.

The effects of RIPC on the induction of cTn levels by an endurance exercise have been studied before (El Messaoudi et al., 2013; Cocking et al., 2017). El Messaoudi et al. (2013) showed that RIPC before 70min of cycling (80% maximal heart rate) and then until exhaustion (95% maximal heart rate) did not affect the cTn levels. On the other hand, Cocking et al. (2017) reported that RIPC before a 1-h time cycling trial led to an attenuation of cTn level increase after the exercise, but without any effect on the left ventricle function. In the current study, while the exercise (running vs. cycling) and load (1 vs. 3.5h) were different from those of the study of Cocking et al. (2017), the outcomes were similar in both studies. A major factor that could explain the different outcome of the study of El Messaoudi et al. (2013) is the amount of muscle tissue involved in the RIPC procedure. In work of El Messaoudi et al. (2013), the RIPC was applied 3×5min bilaterally to the upper arm, while in Cocking et al. (2017), RIPC was applied 4×5min to the ipsilateral upper and lower limb, in an alternating manner. Similar to Cocking et al. (2017), in the current study, the RIPC protocol involved 4×5min intervals of ischemia applied to both lower limbs, which have more muscle mass than the upper limbs. Moreover, lower limbs are mainly involved in tested activities (running, cycling), while upper arms are less involved in cycling. The observed attenuation of the increase of the exercise-induced markers of heart damage after RIPC might indicate its protective effect. However, the questions of whether an exercise-induced increase in cTn levels originates solely in the cardiac muscle and its clinical relevance remain unresolved (Stavroulakis and George, 2020).

To the best of our knowledge, no other study investigating the effects of RIPC on exercise-induced markers of liver damage has been published. However, the data presented in the current study are in agreement with previous reports that participation in the marathon and ultra-marathon distance events results in elevated biomarkers associated with liver injury, including GGT, AST, ALT, and LDH activities (Shin et al., 2016). As most of these enzymes are also present in skeletal muscle, some authors suggest that they reflect skeletal muscle injury rather than liver injury (Lippi et al., 2011). That might be true for other enzymes but not for GGT. First of all, GGT is produced mainly in the liver, with little or no synthesis in skeletal muscle (White et al., 1985). Further, the serum GGT levels in individuals with Duchenne muscular dystrophy are in the normal range despite the substantial skeletal muscle damage indicated by high serum CK levels (Rosales et al., 2008). In the current study, we observed a significant increase in serum CK levels after the marathon run in all participants, indicating skeletal muscle damage. The RIPC training did not significantly affect the serum CK levels. On the other hand, the GGT activity was significantly increased after the run only in the SHAM group, indicating that liver activity during a prolonged strenuous exercise can be successfully impacted by RIPC.

Another marker of liver function that responds to strenuous exercise is bilirubin (De Paz et al., 1995; Shin et al., 2016). In the current study, bilirubin levels increased after the marathon run and returned to baseline over the next 7days. This is in agreement with other studies on strenuous running exercise, including half-marathon (Lippi et al., 2011), marathon (Kratz et al., 2002), and ultramarathon (Fallon et al., 1999; Wu et al., 2004; Arakawa et al., 2016), in which an increase in bilirubin levels was also observed. The increase can be a consequence of hemolysis, which is augmented by endurance running, but also can be a result of impaired liver function in response to exercise (De Paz et al., 1995). Hence, bilirubin, as a catabolite of heme, can be a marker of hepatobiliary insufficiency. While RIPC itself did not affect bilirubin levels at rest, we observed attenuation of BIL-D increase in the RIPC group after the marathon in the current study. Hence, our assumption that RIPC would attenuate exercise-induced increase in bilirubin levels was confirmed. Importantly, low resting bilirubin levels have been associated with increased cardiovascular risk, and exercise training increases resting bilirubin levels (Swift et al., 2012; Kang et al., 2013). However, it is important to make the distinction between training and acute exercise. Collectively, the above data suggest that RIPC protects against exercise-induced liver damage, confirming clinical observations of individuals with liver disease (Rakic et al., 2018).

In eighties of twentieth century, it was demonstrated that contracting skeletal muscles generate free radicals, and that prolonged and intense exercise leads to increased free radical formation and cellular oxidative damage (Davies et al., 1982; Alessio et al., 1988; Reid et al., 1994). Prolonged exercise induces free radical damage of proteins and lipids (Turner et al., 2011; Withee et al., 2017). For example, in one study, levels of MDA, a marker of lipid peroxidation, significantly increased in men after a marathon run (Gomez-Cabrera et al., 2006). We confirmed this observation in the current study, as both CD and MDA levels significantly increased after the run. To the best of our knowledge, this is the first study demonstrating that RIPC training attenuates exercise-induced oxidative stress. The lower level of oxidative stress in RIPC trained runners than that in the SHAM control could also be associated with a lower activity of GGT. The physiological role of GGT is to counteract oxidative stress by breaking down extracellular glutathione and making cysteine available for glutathione synthesis within the cell. Conversely, cysteinylglycine, a product of a GGT-catalyzed reaction, stimulates reactive oxygen species formation and oxidative damage in cell culture in the presence of transferrin iron (Drozdz et al., 1998). This may indicate some contribution of GGT to reactive oxygen species formation during a marathon.

Arterial ketone body ratio is used as an indication of the mitochondrial redox status in hepatic cells (White and Venkatesh, 2011). Decreased AKBR levels are associated with liver dysfunction and are linked to failure of other organs (Shimada et al., 1997). A marked decrease of AKBR levels often leads to a hepatic energy crisis, followed by an impairment of metabolic homeostasis. It is important to note that deleterious changes related to the low AKBR value occur several days after of heart and liver damage (Takahashi et al., 1997). In the current study, the drop in AKBR levels was temporary and the levels rapidly recovered after the marathon run, with a complete return to the baseline value after 1week. Furthermore, the reduction in AKBR levels after the run was much smaller in the RIPC group than that in the SHAM group.

Based on the obtained results, we conclude that repeated RIPC intervention in the form of training exerts a protective effect on the heart and liver by attenuating the induction of exercise-induced markers of organ damage and reducing oxidative stress after a marathon run.

## Data Availability Statement

The raw data supporting the conclusions of this article will be made available by the authors, without undue reservation.

## Ethics Statement

The studies involving human participants were reviewed and approved by Bioethics Committee for Clinical Research at the Regional Medical Chamber in Gdańsk (decision number KB-24/16; Gdańsk, Poland). The patients/participants provided their written informed consent to participate in this study.

## Author Contributions

JM and JA contributed to the conceptualization. JA, JM, AK, BS, BN, AB, LD-S, PB, and KS contributed to the methodology, the writing of the original draft preparation, and writing – review and editing. JM, AB, AK, BS, and BN contributed to the investigation. JM contributed to the project administration. JA contributed to the funding acquisition. All authors contributed to the article and approved the submitted version.

## Author’s Disclaimer

The sponsor had no role in the design of the study; in the collection, analysis, and interpretation of data; in the writing of the manuscript; and in the decision to submit the article for publication. The study was registered as a clinical trial NCT03417700.

## Conflict of Interest

The authors declare that the research was conducted in the absence of any commercial or financial relationships that could be construed as a potential conflict of interest.

## Publisher’s Note

All claims expressed in this article are solely those of the authors and do not necessarily represent those of their affiliated organizations, or those of the publisher, the editors and the reviewers. Any product that may be evaluated in this article, or claim that may be made by its manufacturer, is not guaranteed or endorsed by the publisher.

## Supplementary Material

Supplementary Table 1 Summary of inclusion and exclusion criteria during medical examination.

Supplementary Table 2 Summary of training loads during normal training week in two different training periods (*n*=24).
