# Supplementary material for: Remote Ischemic Preconditioning Reduces Marathon-Induced Oxidative Stress and Decreases Liver and Heart Injury Markers in the Serum
Source: Front Physiol. 2021 Sep 6;12:731889. doi: 10.3389/fphys.2021.731889 (PMC8450527; doi:10.3389/fphys.2021.731889)
Supplement: Supplementary Table 1 — Summary of inclusion and exclusion criteria during medical examination. [file Table_1.pdf]

Supplementary materials

Supplementary Table 1 | Summary of inclusion and exclusion criteria during medical examination

**Inclusion criteria**	1. Aged not less than 30 years and not older than 40
	2. Experienced marathon runner (minimum five starts in full marathon run)
	3. Completion time less than 3 h 20 min
	4. No additional drug intake or smoking and good health status
	5. No additional supplementation
**Exclusion criteria**	1. Physically or mentally illness
	2. Previsions heart and liver problems
	3. Comorbidities causing severe inflammation: asthma, allergy, asthma, celiac disease, psoriasis, Raynaud's disease, rheumatoid arthritis, systemic lupus erythematosus, diabetes, and other.
