# Supplementary material for: Remote Ischemic Preconditioning Reduces Marathon-Induced Oxidative Stress and Decreases Liver and Heart Injury Markers in the Serum
Source: Front Physiol. 2021 Sep 6;12:731889. doi: 10.3389/fphys.2021.731889 (PMC8450527; doi:10.3389/fphys.2021.731889)
Supplement: Supplementary Table 2 — Summary of training loads during normal training week in two different training periods (n=24). [file Table_2.pdf]

Supplementary Table 2 | Summary of training loads during normal training week in two different training periods (n = 24)

		Number of training units per week	CR 1 (km)	CR 2 (km)
General preparation period	Mean	4.85	71.94	16.63
	SD	0.92	13.23	4.22
Pre-start preparation period	Mean	5.4	69.45	18.62
	SD	0.64	12.45	4.22

Note:

CR 1 - 70–80% HR max - continuous running in the first intensity range (70-80% HRmax);

CR 2 - 80–90% HR max - continuous running in the second intensity range (80-90% Hrmax);
